# Combined Effects of Amino Acids in Garlic and Buna-Shimeji (*Hypsizygus marmoreus)* on Suppression of CCl_4_-Induced Hepatic Injury in Rats

**DOI:** 10.3390/foods10071491

**Published:** 2021-06-27

**Authors:** Yusuke Yamaguchi, Yushi Hirata, Takeshi Saito, Hitomi Kumagai

**Affiliations:** 1 Department of Chemistry and Life Science, Nihon University, 1866 Kameino, Fujisawa-shi 252-0880, Japan; yamaguchi.yusuke@nihon-u.ac.jp (Y.Y.); ihsuy0513@gmail.com (Y.H.); 2 ACERA Co., Ltd., 156 Nishitakahashi-machi, Kofu-shi 400-0826, Japan; t-saito@acera-jp.com

**Keywords:** garlic, mushroom, *Hypsizygus marmoreus*, buna-shimeji, ACSO, alliin, arginine, ornithine, hepatic injury

## Abstract

The combination of the garlic-derived amino acid, *S*-allyl-l-cysteine sulfoxide (ACSO), and ornithine or arginine on CCl_4_-induced hepatic injury was examined. After investigating the effectiveness of the mixture of ACSO and ornithine or arginine in preventing hepatic injury in vivo, an extract rich in ACSO and ornithine was prepared by converting arginine in garlic to ornithine by arginase from *Hypsizygus marmoreus* (buna-shimeji), after screening the productivity of ornithine among 12 kinds of mushrooms. Co-administration of ACSO with ornithine or arginine suppressed the increase in aspartate transaminase, alanine transaminase, and thiobarbituric acid reactive substance, and the decrease in glutathione *S*-transferase and cytochrome p450 2E1 activities after CCl_4_ injection more effectively than a single administration of ACSO. All extracts prepared from garlic and buna-shimeji with low and high contents of ACSO and arginine or ornithine significantly suppressed CCl_4_-induced hepatic injury in rats. Considering that ACSO is tasteless, odourless, and enhances taste, and ornithine has a flat or sweet taste and masks bitterness, the extract rich in ACSO and ornithine from garlic and buna-shimeji could be considered a potential antioxidant food material that can be added to many kinds of food to prevent hepatic injury.

## 1. Introduction

Oxidative stress is highly related to diseases such as cancer, vascular diseases, and Alzheimer’s disease [1,2,3]. Excessive amounts of oxidative stress-inducing agents generated in vivo, such as hydroxyl radicals, superoxide anion radicals, and hydrogen peroxide, should be quenched with antioxidative and detoxifying compounds for the prevention of such diseases [4,5,6]. Food and its components, such as phytochemicals [5,7], vitamin E [8], and others [9,10], which play a role in the defence against oxidative stress, have attracted significant attention. These food materials could be effective in preventing oxidative stress-induced diseases.

Garlic has been widely used as a medicinal herb and is a promising food material against oxidative stress [11]. The major medicinal components in garlic are diallyl disulphide (DADS) and diallyl trisulfide (DATS) [12,13,14,15,16,17], which increase antioxidant activity [18,19,20,21,22]. When garlic is crushed or sliced, these sulphides, which are responsible for the characteristic garlic odour, are produced from an amino acid, *S*-allyl-l-cysteine sulfoxide (ACSO; also known as alliin), by cysteine-*S*-conjugate β-lyase (C-S lyase), followed by spontaneous reactions of sulfenic acid [23]. Although these sulphides show potent antioxidant effects, it is difficult to use them as food materials because they are volatile, hydrophobic, and have a strong odour. In contrast, ACSO, a dominant amino acid in raw garlic, is non-volatile, hydrophilic, odourless, tasteless, and a taste enhancer [24]. It can be used in many kinds of food without impairing palatability. We have already shown that ACSO has antioxidant activity, induces antioxidative and detoxifying enzymes, and suppresses hepatic injury in rats [25]. Another dominant amino acid in garlic is arginine, which is hydrolysed to ornithine by arginase. Although there is little scientific evidence that ornithine alleviates hepatic injury despite the common assumption of its function in Japan, ornithine aspartate, a stable salt, reportedly shows hepatoprotective activity against thioacetamide-induced hepatopathy [26].

Some mushrooms have highly active arginase that catalyses the hydrolysis of arginine to ornithine and urea, accumulating a considerable amount of urea in the fruit bodies to regulate osmotic pressure [27]. Thus, an extract rich in ACSO and ornithine could be prepared by mixing garlic extracts with inactivated C-S lyase and mushroom extracts with arginase. In addition, as compounds in the urea cycle that convert toxic ammonia to urea, arginine and ornithine may protect hepatocytes via a different mechanism from ACSO. Therefore, the concomitant use of ACSO and arginine or ornithine may more effectively prevent hepatic injury induced by toxic agents, such as CCl_4_ and ammonia, than the single use of ACSO.

In this study, the protective effect of co-administration of synthesised ACSO with ornithine or arginine against CCl_4_-induced hepatic injury was investigated. We then screened mushrooms that effectively converted arginine to ornithine. After selecting the mushroom, the garlic extract with low or high ACSO content was mixed with the mushroom extract containing arginase to convert arginine to ornithine. In addition, a garlic extract with a low or high ACSO content not mixed with the mushroom extract was prepared to examine the effect of arginine. The antioxidant effects of these extracts were evaluated in terms of the suppression of CCl_4_-induced hepatic injury, which has been well studied and is suitable for the evaluation of antioxidative and detoxifying effects of food materials [28,29,30,31]. This study proposes a novel food combination to present a potent antioxidative effect.

## 2. Materials and Methods

### 2.1. Materials

ACSO was chemically synthesised according to previous reports [25,32]. l-Arginine was purchased from Daesang Co., Ltd. (Seoul, Korea). l-Ornithine was purchased from Kyowa Hakko Bio Co., Ltd. (Tokyo, Japan). Other chemical reagents used in this study were purchased from FUJIFILM Wako Pure Chemical Corporation (Osaka, Japan), Kanto Chemical Co., Inc. (Tokyo, Japan), Oriental Yeast Co., Ltd. (Tokyo, Japan), Cosmobio Co., Ltd. (Tokyo, Japan), Nacalai Tesque, Inc. (Kyoto, Japan), and Roche Diagnostics GmbH Co., Ltd. (Mannheim, Germany).

### 2.2. Screening of Mushrooms with High Ornithine Production

Twelve kinds of mushrooms, namely, Hypsizygus marmoreus (buna-shimeji), Pleurotus eryngii, Agaricus blazei, Flammulina velutipes, Pleurotus eryngii var. tuoliensis, Pleurotus ostreatus, Hericium erinaceus, Pleurotus cornucopiae, Agaricus bisporus, Lyophyllum decastes, Lentinula edodes, and Grifola frondosa, were purchased from a local market. Frozen mushrooms were homogenised in deionised water of the same weight using a household juicer. The homogenate was centrifuged at 3000× g for 10 min, and the supernatant was collected. The supernatant (20 mL) was mixed with 0.4 g of arginine and adjusted to pH 10 using 1 M NaOH. The mixture was stirred at 50 °C for 8 h. The ornithine, arginine, urea, and ammonia concentrations in the resultant supernatant were measured using an amino acid analyser (JLC-500/V, JEOL Ltd. Tokyo, Japan).

### 2.3. Preparation of Garlic Extract, Garlic Plus Buna-Shimeji Extract, Heated-Garlic Extract, and Heated-Garlic Plus Buna-Shimeji Extract

Peeled garlic obtained from a local market was chopped with a household food chopper (MK-K78, Panasonic, Osaka, Japan) and homogenised using a homogeniser (Ace homogenizer, Nihonseikikaisha Ltd., Tokyo, Japan). The homogenate was stirred in 75% ethanol, and the solution was centrifuged at 3000× *g.* The supernatant was lyophilised, and the dried residue was designated as the garlic extract (Ge).

The pileus of buna-shimeji obtained from a local market was frozen. Frozen pileus and Ge were mixed and homogenised. The pH of the homogenate was adjusted to 9.5 with 1 M NaOH. The homogenate was kept for 16 h at 40 °C to convert arginine to ornithine. After the conversion was confirmed using an amino acid analyser, 75% ethanol was added to the homogenate and stirred for an hour. The solution was centrifuged at 3000× *g*, and the supernatant was concentrated under vacuum to remove ethanol, followed by neutralisation using citric acid. The dried residue after lyophilisation of the neutralised supernatant was designated as garlic plus buna-shimeji extract (GBe).

Peeled garlic obtained from the local market was heated in hot water at 80 °C for 30 min. The heated garlic was cooled to room temperature (ca. 25 °C), chopped, and homogenised. The homogenate was stirred in 75% ethanol, and the solution was centrifuged at 3000× *g.* The supernatant was concentrated under vacuum to remove ethanol and lyophilised. The dried residue was designated as heated-garlic extract (HGe).

Heated-garlic plus buna-shimeji extract (HGBe) was prepared by replacing Ge with HGe in the same way used for the preparation of GBe.

The ACSO content was determined using high performance liquid chromatography (HPLC) after derivatisation with *o*-phthalaldehyde. The contents of l-arginine, l-ornithine, and other amino acids were determined using an amino acid analyser.

### 2.4. Animals

Five-week-old male Sprague Dawley rats were purchased from Japan SLC, Inc. (Tokyo, Japan). Experiments on the animals were performed in accordance with the Guidelines for Animal Experiments of the College of Bioresource Sciences, Nihon University (approval code: AP13B010). The rats were housed in individual, stainless-steel, wire cages with free access to food (CE-2, Clea Japan, Tokyo, Japan) and water during a seven-day acclimation period before the experiments. The feeding facility was maintained at approximately 21 °C with a 12 h light-dark cycle.

### 2.5. Effect of Co-Administration of ACSO with Arginine or Ornithine on Suppression of Hepatic Injury Induced by CCl_4_

The rats were randomly divided into eight groups of six rats each. ACSO was dissolved in distilled water and orally administered via gavage at a dosage of 100 μmol/mL/day for five consecutive days. A mixture of 50 μmol of ACSO and 50 μmol of l-ornithine or l-arginine in 1 mL of distilled water was orally administered via gavage for five consecutive days in the ACSO + arginine (ACSO + Arg) or ACSO + ornithine (ACSO + Orn) group, respectively. The distilled water (DW) group received only 1 mL of distilled water per day via gavage for five consecutive days. CCl_4_ was intraperitoneally administered at a dosage of 0.5 mL/kg body weight after the 5th oral administration of samples in the CCl_4_ groups. The rats were then subjected to fasting for 18 h, and their body weight was measured. The blood was collected, treated with sodium citrate, and centrifuged at 1500× *g* for 15 min at 4 °C to obtain the serum for further experiments. The liver was excised, and its weight was measured. The microsomal and cytosolic fractions were prepared by centrifugation, as described previously [33]. These fractions were used in further experiments.

### 2.6. Effect of GBe on Suppression of Hepatic Injury Induced by CCl_4_

The rats were randomly divided into 10 groups of six rats each. Briefly, 10% of Ge, GBe, HGe, and HGBe were dissolved in distilled water, and the solution was orally administered at a dosage of 1 mL/day via gavage for five consecutive days to the corresponding group. The DW group received only 1 mL of distilled water per day via gavage for five consecutive days. CCl_4_ was intraperitoneally administered at a dosage of 0.5 mL/kg body weight after the 5th oral administration of samples in the CCl_4_ groups. The remaining procedure was the same as that described in Section 2.5.

### 2.7. Determination of Aspartate Transaminase and Alanine Transaminase Levels

The serum obtained was used to determine aspartate transaminase (AST) and alanine transaminase (ALT) levels using an enzymatic method with an automatic analyser, Spotchem EZ SP-4430 (Liver-2, Arkray, Inc., Kyoto, Japan).

### 2.8. Measurement of CYP2E1 Activity

The activity of cytochrome p450 2E1 (CYP2E1) was determined according to a previously described method [34]. Briefly, the microsome fraction of the liver was incubated at 37 °C for 30 min in a potassium phosphate buffer containing nicotinamide adenine dinucleotide phosphate (NADP), d-glucose-6-phosphate, magnesium chloride, glucose-6-phosphate dehydrogenase, sodium citrate, and *p*-nitrophenol. Then, trichloroacetic acid was added to the solution and cooled in an ice bath. The mixture was centrifuged, and the supernatant was collected. The supernatant was mixed with 2 M NaOH, and the absorbance at 535 nm was measured to determine the amount of *p*-nitrocatechol produced by the oxidation of *p*-nitrophenol by CYP2E1. The protein concentration of the microsome fraction of the liver was determined using the bicinchoninic-acid assay.

### 2.9. Measurement of Lipid Peroxide

The lipid peroxide content in the liver was determined using a thiobarbituric acid-reactive substance (TBARS) assay, according to a previous report [25]. Briefly, the liver homogenate was mixed with 1% phosphoric acid and 0.67% thiobarbituric acid (TBA) solution. The mixture was incubated at 95 °C for 45 min, and then aldehyde-TBA adduct was extracted using *n*-butanol. The absorbance of aldehyde-TBA adduct at 535 nm was measured. Malondialdehyde (MDA) was used as a standard, and the TBARS value was expressed as MDA equivalent.

### 2.10. Measurement of Glutathione S-Transferase Activity

The activity of glutathione *S*-transferase (GST) in the cytosol fraction of the liver was assayed spectrophotometrically according to a previous report [25]. Briefly, the cytosol fraction from the liver and 1-chloro-2,4-dinitrobenzene (CDNB) were added to a solution containing 30 mM reduced glutathione (GSH) in 0.1 M potassium phosphate buffer at pH 7.4 at 25 °C. The increase in the absorbance at 340 nm attributed to *S*-2,4-dinitrophenylglutathione was measured. The activity of GST was expressed as the amount of *S*-2,4-dinitrophenylglutathione produced per minute per milligram of cytosol protein. The protein concentration of the cytosol fraction of the liver was determined using the bicinchoninic-acid assay.

### 2.11. Statistical Analysis

All data are expressed as mean ± standard deviation (SD), and the significance of the differences between groups was evaluated using a one-way analysis of variance followed by Tukey–Kramer’s test.

## 3. Results

### 3.1. Effect of Co-Administration of Arginine or Ornithine with ACSO on CCl_4_-Induced Hepatic Injury

**Figure 1:**
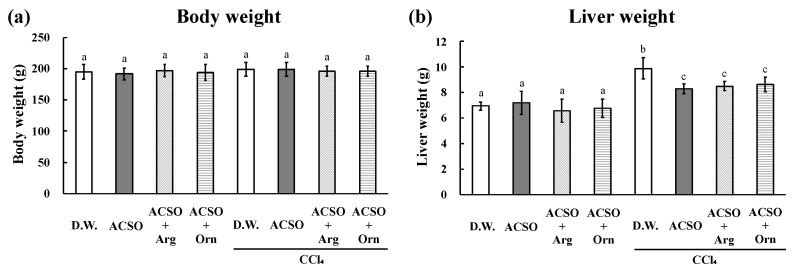
Effect of co-administration of arginine or ornithine with ACSO on body weight (**a**), and the liver weight (**b**) of rats with CCl_4_-induced hepatic injury. Six rats in each group received a sample (ACSO, ACSO + Arg, or ACSO + Orn) orally for 5 consecutive days. CCl_4_ was intraperitoneally injected, then body weight and liver weight were measured. Co-administration of arginine or ornithine with ACSO suppressed the increase in the liver weight. Each value represents the mean ± standard deviation (SD). The different letters with bars in the figures indicate a significant difference between the groups (*p* < 0.01). The same letters with bars indicate no significant difference between the groups (*p* < 0.01).

**Figure 2:**
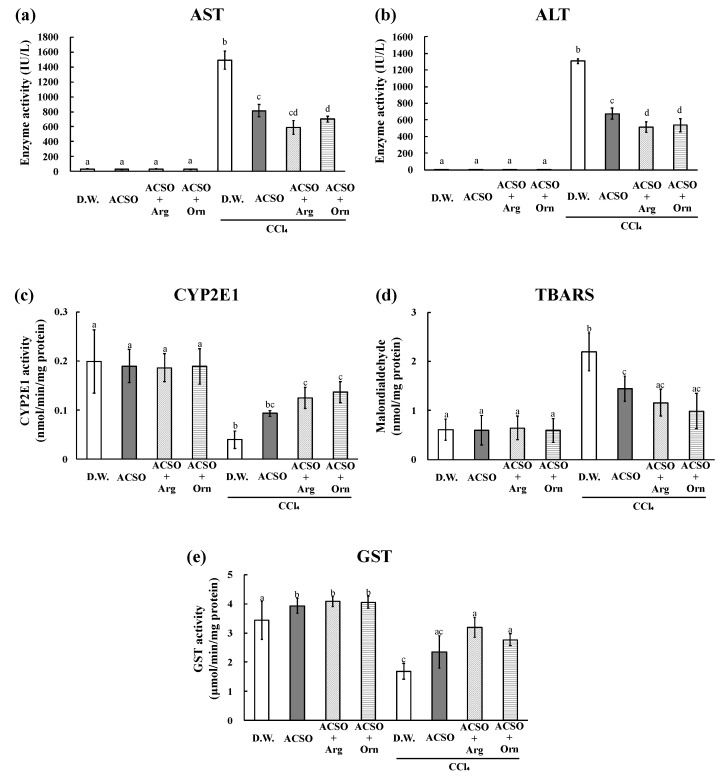
Effect of co-administration of arginine or ornithine with ACSO on aspartate transaminase (AST) activity (**a**), alanine transaminase (ALT) activity (**b**), cytochrome p450 2E1 (CYP2E1) activity (**c**), amount of thiobarbituric acid reactive substances (TBARS) (**d**), and glutathione-*S*-transferase (GST) activity (**e**) in rats with CCl_4_-induced hepatic injury. Six rats in each group received a sample (ACSO, ACSO + Arg, or ACSO + Orn) orally for 5 consecutive days. CCl_4_ was intraperitoneally injected, and the blood and the liver were collected to assess the degree of hepatic injury. Co-administration of arginine or ornithine with ACSO suppressed the increases in AST and ALT levels more than a single administration of ACSO. Each value represents the mean ± SD. The different letters with bars in figures (**a**–**d**) indicate a significant difference between the groups (*p* < 0.01). The different letters with bars in figure (**e**) indicate a significant difference between the groups (*p* < 0.05). The same letters indicate no significant difference between the groups in figures (**a**–**d**) at *p* < 0.01 and in figure (**e**) at *p* < 0.05.

The liver weight, AST and ALT activities, and TBARS increased, whereas CYP2E1 and GST activities decreased in the DW group after the intraperitoneal injection of CCl_4_, which are typical changes observed in rats with CCl_4_-induced hepatic injury (Figure 1 and Figure 2). These changes induced by CCl_4_ were significantly suppressed in the ACSO, ACSO + Arg, and ACSO + Orn groups, even after CCl_4_ injection (*p* < 0.01). AST and ALT activities and TBARS in the ACSO + Arg and ACSO + Orn groups treated with CCl_4_ were lower than those in the ACSO group treated with CCl_4_. CYP2E1 and GST activities in the ACSO + Arg and ACSO + Orn groups treated with CCl_4_ were higher than those in the ACSO group treated with CCl_4_. In particular, AST activity of the ACSO + Orn group treated with CCl_4_ was significantly lower than that of the ACSO group treated with CCl_4_ (*p* < 0.01). ALT activity of the ACSO + Arg and ACSO + Orn groups treated with CCl_4_ was significantly lower than that of the ACSO group treated with CCl_4_ (*p* < 0.01). In the absence of CCl_4_ administration, GST activity in the ACSO, ACSO + Arg, and ACSO + Orn groups was significantly higher than that in the DW group (*p* < 0.05).

### 3.2. Ornithine Production by Mushrooms

**Figure 3:**
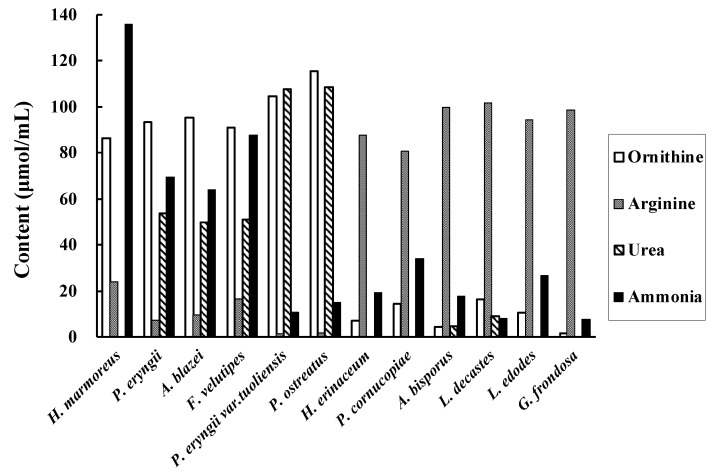
Ornithine, arginine, urea, and ammonia concentrations after arginine addition in the extracts of 12 kinds of mushrooms.

The ornithine, arginine, urea, and ammonia contents in the mushroom extract after arginine addition are shown in Figure 3. *H. marmoreus*, *P. eryngii*, *A.blazei, F. velutipes*, *P. eryngii var. tuoliensis*, and *P. ostreatus* showed high ornithine concentration (86, 93, 95, 91, 105, and 116, μmol/mL, respectively) in the extract. The ornithine concentration in these solutions before arginine addition was approximately 4 μmol/mL. Urea was not detected in *the H. marmoreus* extract. As ammonia can be removed by lyophilisation during sample preparation, we decided to use *H. marmoreus* to convert arginine in garlic.

### 3.3. Contents of Amino Acids including ACSO, Arginine, and Ornithine in Ge, GBe, HGe, and HGBe

Among the three compounds, ACSO, arginine, and ornithine, Ge predominantly contained arginine, while GBe predominantly contained ornithine (Table 1). On the other hand, HGe primarily contained both ACSO and arginine, while HGBe primarily contained both ACSO and ornithine. The contents of all the other amino acids were lower than 1% in Ge, Gbe, HGe, and HGBe (Table S1). The contents of aspartic acid, cystine, methionine, and tyrosine, reported to suppress CCl_4_-induced hepatic injury [35], were much lower than those of ACSO, arginine, and ornithine in the extracts.

### 3.4. Effect of Ge, Gbe, HGe, and HGBe on CCl_4_-Induced Hepatic Injury

**Figure 4:**
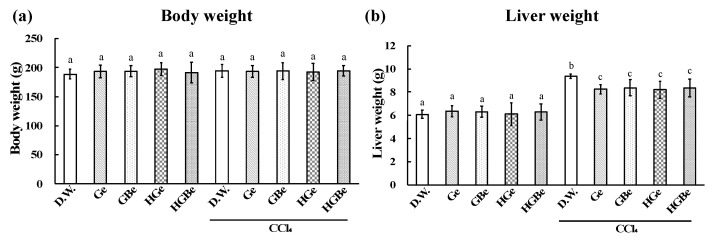
Effect of Ge, GBe, HGe, and HGBe on body weight (**a**) and liver weight (**b**) of rats with CCl_4_-induced hepatic injury. Six rats in each group received a sample (Ge, GBe, HGe, or HGBe) orally for 5 consecutive days. CCl_4_ was intraperitoneally injected, then body weight and the liver weight were measured. The extract of garlic and garlic with buna-shimeji suppressed the increase in liver weight. Each value represents the mean ± SD. The different letters with bars in the figures indicate a significant difference between the groups (*p* < 0.05). The same letters with bars indicate no significant difference between the groups (*p* < 0.05).

**Figure 5:**
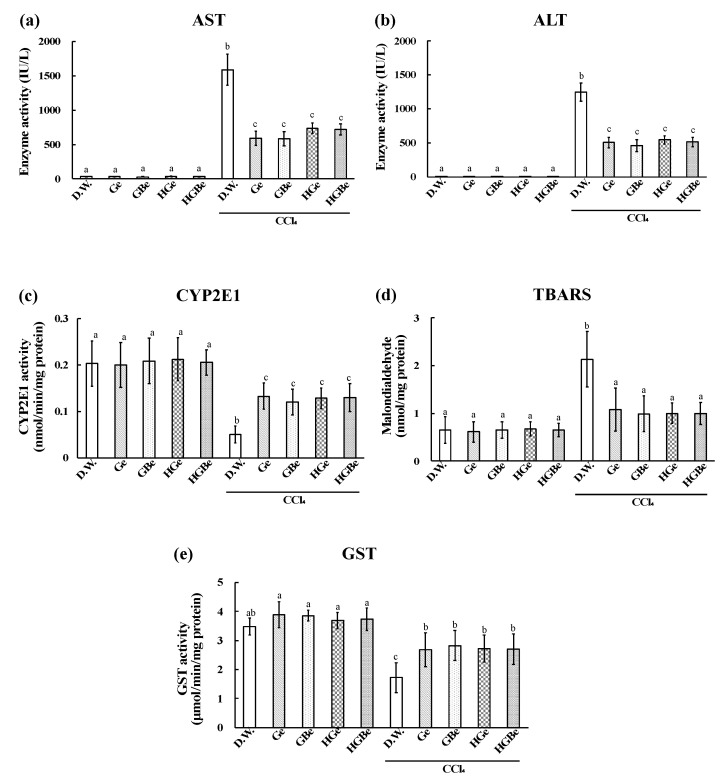
Effect of Ge, GBe, HGe, and HGBe on aspartate transaminase (AST) activity (**a**), alanine transaminase (ALT) activity (**b**), cytochrome p450 2E1 (CYP2E1) activity (**c**), amount of thiobarbituric acid reactive substances (TBARS) (**d**), and glutathione-*S*-transferase (GST) activity (**e**) in rats with CCl_4_-induced hepatic injury. Six rats in each group received an extract (Ge, GBe, HGe, or HGBe) orally for 5 consecutive days. CCl_4_ was intraperitoneally injected, and the blood and the liver were collected to assess the degree of hepatic injury. The extract of garlic and garlic with buna-shimeji suppressed the increases in AST, ALT, and TBRAS levels and the decreases in CYP2E1 and GST levels. Each value represents the mean ± SD. The different letters with bars in figure (**d**) indicate a significant difference between the groups (*p* < 0.01). The different letters with bars in the figures (**a**–**c**,**e**) indicate a significant difference between the groups (*p* < 0.05). The same letters with bars indicate no significant difference between the groups in figure (**d**) at *p* < 0.01 and in figures (**a**–**c**,**e**) at *p* < 0.05.

The elevation in liver weight, AST, ALT, and TBARS induced by CCl_4_ injection was significantly suppressed in the Ge, GBe, HGe, and HGBe groups (*p* < 0.05, Figure 4 and Figure 5). The decrease in CYP2E1 and GST activities induced by CCl_4_ injection was also suppressed in the Ge, GBe, HGe, and HGBe groups (*p* < 0.05). GST activities in the Ge, GBe, HGe, and HGBe groups were higher than those in the DW group in the absence of CCl_4_ administration, but the difference was not considered significant.

## 4. Discussion

Garlic is known to have various physiological functions, and sulphides such as DADS and DATS are considered to be the major active components of garlic. In our previous study, we showed that ACSO, an odour precursor, also has various functions, such as prevention of CCl_4_-induced hepatic injury, inhibition of platelet aggregation, and suppression of the increase in blood ethanol levels [25,36,37].

After intraperitoneal injection, CCl_4_ is reduced by cytochrome P450 2E1 (CYP2E1) to the CCl_3_ radical in the liver [38,39], which forms covalent bonds with proteins, nucleic acids, and lipids, thus impairing their functions [40,41]. Moreover, CCl_3_ radicals oxidise lipids to produce lipid peroxide, in addition to destroying the lipid bilayer of cell membranes, resulting in leakage of the contents of the liver cells, such as AST and ALT, into the blood [42]. Lipid peroxide is reduced by glutathione (GSH) or conjugated with GSH by GST and excreted. Thus, the preventive effect on CCl_4_-induced hepatic injury was evaluated based on AST and ALT activities in the blood, CYP2E1 and GST activities, and lipid peroxide value in the liver after oral administration of samples following intraperitoneal injection of CCl_4_. The injection of CCl_4_ significantly increased AST and ALT activities and TBARS values but decreased CYP2E1 and GST activities. However, co-administration of arginine/ornithine with ACSO and a single administration of ACSO effectively suppressed hepatic injury (Figure 2).

One of the mechanisms for preventing CCl_4_-induced hepatic injury by ACSO is the promotion of nuclear translocation of NF-E2-related factor 2 (Nrf2) to induce antioxidative and detoxifying enzymes [25]. Arginine suppresses oxidative stress by promoting Nrf2 translocation to the nucleus [43]. In contrast, ornithine aspartate lowers the blood ammonia concentration [44], which is considered to be one of the reasons for its hepatoprotective activity [26]. In addition, the administration of ornithine aspartate suppressed the decrease in liver tissue GSH level caused by thioacetamide-induced hepatopathy [26]. Ornithine may also function as a suppressor of oxidative stress caused by CCl_4_, probably owing to the biosynthesis of GSH via glutamate [45]. As a compound in the urea cycle that is converted to ornithine, arginine may have worked in a way similar to ornithine. Although the dose of ornithine aspartate previously [26] was approximately 200 μmol, we administered 50 μmol of ornithine/arginine together with 50 μmol of ACSO in 1 mL of distilled water to rats for five consecutive days before intraperitoneal injection of CCl_4_ and compared the effect with that of 100 μmol of ACSO. Although the dose of ornithine/arginine in this study was lower than that of ornithine aspartate (200 μmol) in the literature [26], and the dose of ACSO in the ACSO + Orn and ACSO + Arg groups was lower than that in the ACSO group, the co-administration of 50 μmol of ornithine/arginine with 50 μmol of ACSO suppressed the increase in AST and ALT activities more potently than the administration of 100 μmol ACSO. This result indicates that the combination of ornithine/arginine and ACSO showed a synergistic effect. One of the reasons for this synergistic effect might be the nuclear translocation of Nrf2 by ACSO and arginine and the elimination of toxic ammonia by ornithine/arginine.

Some mushrooms are considered to have arginase, which converts arginine to ornithine and urea, and urease, which hydrolyses urea to ammonia and carbon dioxide. Therefore, to examine the activities of arginase and urease in mushrooms, urea, ammonia, ornithine, and arginine contents in mushroom extracts were measured after the addition of arginine and incubation at 50 °C for 8 h. Among the mushrooms examined, *H. marmoreus*, *P. eryngii*, *A. blazei, F. velutipes*, *P. eryngii var. tuoliensis*, and *P. ostreatus* showed high ornithine levels, indicating high arginase activity. *H. marmoreus* contained no urea; nonetheless, it contained the largest amount of ammonia, indicating that it also has high urease activity. Urea is difficult to remove from the extract, and the remaining urea may disturb the effective conversion of arginine to ornithine. In contrast, ammonia can easily be removed by lyophilisation. Therefore, we chose *H. marmoreus* as the source of arginase for the conversion of arginine in the garlic extracts.

The ACSO content in Ge and GBe was 4.5 and 7.3 μmol/g-extract, respectively, while that in HGe and HGBe was 81.8 and 65.5 μmol/g-extract, respectively. This result indicates that C-S lyase was effectively inactivated by heating garlic at 80 °C for 30 min. The arginine content in Ge and HGe was 183.1 and 181.4 μmol/g-extract, respectively, while the ornithine content in GBe and HGBe was 127.9 and 149.1 μmol/g-extract, respectively. Therefore, approximately 70–80% of arginine is converted to ornithine by arginase in *H. marmoreus*.

As 10% of Ge, GBe, HGe, and HGBe in 1 mL of distilled water was orally administered to rats, the dose of ACSO in the Ge, GBe, HGe, HGBe groups was 0.45, 0.73, 8.18, and 6.55 μmol/day (Table 2), respectively, which was approximately 1/100 to 1/10 the dose of synthesised ACSO in the former experiment. In contrast, the dose of arginine in the Ge and HGe groups was 18.31 and 18.14 μmol, respectively, while that of ornithine in the GBe and HGBe groups was 12.79 and 14.91, respectively. Although the doses of arginine and ornithine in these groups were approximately 1/3 the dose in the former experiment, the hepatic injury was significantly suppressed in all the groups (Ge, GBe, HGe, and HGBe groups). Therefore, even less than 20 μmol of arginine and ornithine would be effective to exert their function in the presence of ACSO and some other compounds in the extract. As volatile sulphides such as DADS and other amino acids that suppress CCl_4_-induced hepatic injury could be negligible in all garlic extracts, ACSO, arginine, and ornithine would be the major factors responsible for this effect.

The taste of bioactive compounds is important for their use in functional foods. ACSO is tasteless, odourless, and reportedly enhances the taste of food [24]. Arginine has a bitter taste [46], whereas ornithine has a flat or sweet taste [47,48]. Moreover, ornithine masks the bitter taste better than arginine [48]. Although precise mechanisms should be clarified for the hepatoprotective effect by multi-components, the conversion of arginine to ornithine in garlic using mushroom extract could offer a food material that protects the liver and improves the palatability of food.

## 5. Conclusions

The co-administration of ornithine/arginine with ACSO suppressed CCl_4_-induced hepatic injury in rats more potently than a single administration of ACSO, probably owing to synergistic effects. The extract with high contents of ACSO and ornithine was prepared from garlic and *H. marmoreus* (buna-shimeji), which converted arginine in garlic to ornithine by arginase. All garlic extracts with low and high ACSO contents and with arginine or ornithine alleviated CCl_4_-induced hepatic injury in rats. Considering that ornithine has a flat or sweet taste and masks bitter taste, the extract with high contents of ACSO and ornithine can be used as an antioxidant food material to protect against hepatic injury.

## Figures and Tables

**Table 1:** Contents of ACSO, arginine, and ornithine in garlic and bunashimeji extracts.

Group	ACSO (%)	Arginine (%)	Ornithine (%)
Ge	0.08	3.19	0.02
GBe	0.13	0.04	1.69
HGe	1.45	3.16	0.03
HGBe	1.16	0.01	1.97

**Table 2:** Daily dose of ACSO, arginine, and ornithine in the animal experiments.

Group	ACSO (μmol)	Arginine (μmol)	Ornithine (μmol)
Ge	0.45	18.31	0.15
GBe	0.73	0.23	12.79
HGe	8.18	18.14	0.23
HGBe	6.55	0.06	14.91

## Data Availability

The data presented in this study are available on request from the corresponding author. The data are not publicly available due to their large volume and little interest.

## Supplementary Materials

The following are available online at https://www.mdpi.com/article/10.3390/foods10071491/s1, Table S1: Contents of amino acids in garlic and bunashimeji extracts.
