# Supplementary material for: Combined Effects of Amino Acids in Garlic and Buna-Shimeji (Hypsizygus marmoreus) on Suppression of CCl4-Induced Hepatic Injury in Rats
Source: Foods. 2021 Jun 27;10(7):1491. doi: 10.3390/foods10071491 (PMC8306630; doi:10.3390/foods10071491)
Supplement: Supplementary file 1 [file foods-10-01491-s001.zip › foods-1243990-supplementary.pdf]

Table S1 Contents of amino acids in garlic and bunashimeji extracts.

Amino Acids (mg/100 g)	Ge	Gbe	HGe	HGBe
Asparaginic Acid	18.1	30.4	N. D.	N. D.
Threonine	30.9	125.3	28.9	97.1
Serine	32.5	148.1	32.7	153.9
Aspargine	241.0	149.1	214.2	156.5
Glutamic Acid	143.8	422.5	150.4	409.0
Glutamine	15.0	122.5	19.2	119.6
Glycine	15.3	106.6	20.6	121.0
Alanine	284.0	569.8	198.5	491.7
Valine	N. D.	N. D.	N. D.	N. D.
Cystine	N. D.	N. D.	N. D.	N. D.
Methionine	N. D.	N. D.	6.3	40.2
Isoleucine	12.6	89.0	8.0	86.1
Leucine	18.0	170.1	20.3	187.2
Tyrosine	3.0	73.8	4.3	77.4
Phenylalanine	31.7	86.7	30.3	125.3
Histidine	N. D.	5.7	10.6	15.0
Lysine	213.7	263.3	267.2	375.7
Tryptophan	N. D.	N. D.	N. D.	N. D.
Arginine	**3189.2**	**44.2**	**3163.6**	**7.4**
Proline	80.1	153.2	79.3	167.3
Phosphoserine	56.6	87.6	11.4	27.3
Taurine	N. D.	N. D.	N. D.	N. D.
Phosphoethanolamine	N. D.	N. D.	N. D.	N. D.
Urea	N. D.	730.9	N. D.	54.5
Sarcosine	N. D.	N. D.	N. D.	N. D.
α-Aminoadipic Acid	N. D.	N. D.	N. D.	N. D.
Citrulline	32.6	39.8	29.4	50.2
α-Aminobutyric Acid	N. D.	2.3	N. D.	37.6
Cystathionine	2.8	49.3	86.6	109.8
β-Alanine	N. D.	40.8	N. D.	75.7
β-Aminobutyric Acid	N. D.	N. D.	N. D.	N. D.
γ-Aminobutyric Acid	78.4	76.1	82.0	147.5
Monoethanolamine	N. D.	N. D.	N. D.	N. D.
Ammonia	802.1	49.5	123.1	56.6
Hydroxylysine	N. D.	N. D.	N. D.	N. D.
Ornithine	**22.9**	**1693.2**	**25.8**	**1972.4**
1-Methylhistidine	9.0	N. D.	5.6	N. D.
3-Methylhistidine	N. D.	N. D.	N. D.	N. D.
Anserine	N. D.	N. D.	N. D.	N. D.
Carnosine	N. D.	N. D.	N. D.	N. D.
Hydroxyproline	N. D.	N. D.	N. D.	N. D.

N.D. not determined
